# Awareness and Knowledge of Necrotizing Fasciitis: A Descriptive Cross-Sectional Study

**DOI:** 10.7759/cureus.95345

**Published:** 2025-10-24

**Authors:** Waqar Farooqi, Saud S Al Mohrij, Abdulrauof O Elemam, Azzam K Almasri, Hessa M Alanazi, Amirah Alnaser, Mohammed Abanmi

**Affiliations:** 1 Internal Medicine, Faculty of Medicine, AlMaarefa University, Riyadh, SAU; 2 College of Medicine, AlMaarefa University, Riyadh, SAU; 3 General Practice, AlMaarefa University, Riyadh, SAU; 4 Medicine, AlMaarefa University, Riyadh, SAU; 5 College of Medicine, King Saud Bin Abdulaziz University for Health Sciences, Riyadh, SAU

**Keywords:** cross-sectional study, group a streptococcus, health education, knowledge assessment, necrotizing fasciitis, public awareness, saudi arabia, skin, soft tissue infection

## Abstract

Background

Necrotizing fasciitis (NF) is a rare but rapidly progressive soft tissue infection associated with significant morbidity and mortality. Early recognition and prompt management are critical to improving outcomes. However, awareness and understanding of the disease among the general population and even healthcare professionals remain limited.

Objective

This study aimed to assess the level of awareness and knowledge of NF among healthcare providers and the general public in Riyadh, Saudi Arabia.

Methods

A cross-sectional study was conducted using a validated, structured questionnaire distributed to 106 participants, including both healthcare workers and members of the public. The questionnaire covered sociodemographic data, general awareness, knowledge of causative organisms, symptom progression, risk factors, and attitudes toward treatment and prevention. Data were analyzed using SPSS version 26 (IBM Corp., Armonk, NY), and associations between demographic variables and knowledge levels were assessed using the chi-square test.

Results

A majority of participants (n = 74, 69.8%) had heard of NF, and 65 (61.3%) correctly identified bacterial infection as the primary cause. Less than half (n = 48, 45.3%) recognized group A *Streptococcus* as a common causative organism, and 47 (44.3%) were aware that symptoms can progress within hours. Knowledge levels were significantly associated with gender (p = 0.001), age (p = 0.009), education level (p = 0.009), and occupation (p = 0.001), with healthcare providers and postgraduate participants demonstrating higher awareness. Attitudes toward early treatment and public education were largely positive, with 75 (70.8%) emphasizing their importance.

Conclusion

Although basic awareness of NF exists among the surveyed population, significant gaps remain in understanding its etiology, rapid progression, and risk factors. Targeted educational interventions are essential to bridge these gaps, particularly among non-healthcare groups, to improve early recognition and reduce delays in treatment.

## Introduction

Necrotizing fasciitis (NF) is a rare but rapidly progressing soft tissue infection characterized by widespread necrosis of the fascia and subcutaneous tissues, most commonly involving the extremities (especially the lower limbs), perineum, and abdominal wall. The condition is often misdiagnosed in its early stages due to its nonspecific symptoms, leading to delayed treatment and increased mortality rates [1]. The disease can occur in individuals of all ages and is often associated with comorbidities such as diabetes mellitus, immunosuppression, and peripheral vascular disease, which can increase susceptibility to infection and worsen clinical outcomes [2]. The causative organisms of NF include group A *Streptococcus*, *Staphylococcus aureus*, *Vibrio vulnificus*, and polymicrobial infections involving anaerobes and gram-negative bacteria. These pathogens release exotoxins and enzymes that facilitate rapid tissue destruction, thrombosis of blood vessels, and systemic inflammatory responses [3]. The aggressive nature of the disease necessitates prompt medical intervention, including broad-spectrum antibiotic therapy, fluid resuscitation, and urgent surgical debridement to remove necrotic tissue and control the infection [4]. Early clinical recognition of NF is critical, as its presentation can often mimic less severe conditions such as cellulitis or simple abscesses. Patients commonly report severe, disproportionate pain, rapid swelling, erythema, and tenderness, with subsequent progression to skin necrosis, hemorrhagic bullae, and systemic toxicity [5]. The systemic manifestations, including fever, hypotension, and altered mental status, reflect the severity of the underlying infection and the risk of septic shock and multiorgan failure if treatment is delayed [6]. Despite advancements in diagnostic tools, including imaging modalities such as computed tomography (CT) and magnetic resonance imaging (MRI), clinical judgment remains paramount in making a timely diagnosis. The Laboratory Risk Indicator for Necrotizing Fasciitis (LRINEC) score has been suggested as a valuable tool for identifying high-risk patients, although its effectiveness is still a topic of debate [7]. The definitive diagnosis is frequently confirmed during surgery, where key findings include grayish necrotic tissue, minimal bleeding, and the presence of foul-smelling discharge [8]. Due to the high morbidity and mortality associated with NF, a multidisciplinary approach involving infectious disease specialists, surgeons, intensivists, and wound care teams is essential for optimizing patient outcomes. Delays in surgical intervention have been linked to increased mortality rates, reinforcing the need for early recognition and aggressive management strategies [9]. Furthermore, patient education and preventive strategies, including proper wound care and early medical consultation for suspicious soft tissue infections, play a critical role in reducing disease burden and improving survival rates [10,11].

## Materials and methods

Study design

This was a cross-sectional, self-administered survey-based study designed to assess awareness and knowledge of NF among healthcare professionals and the general public in Riyadh, Saudi Arabia, with a total sample size of 106 participants.

Study setting

The study was conducted in community centers across Riyadh, targeting healthcare providers and the general public.

Eligibility criteria

Inclusion criteria included healthcare professionals and members of the general public aged 18 years and above who were willing to participate. Exclusion criteria included participants below 18 years of age.

Data collection

Data were gathered using structured online questionnaires in Arabic and English. The questionnaire assessed participants' baseline knowledge, understanding of NF symptoms, risk factors, and management approaches.

Instruments and validation

The first section of the questionnaire consisted of sociodemographic data, including gender, age, nationality, marital status, education level, and occupation. The second section focused on assessing participants’ awareness and knowledge of NF. This section included multiple questions regarding the cause, common bacterial agents, symptom progression, risk factors, and the possibility of NF occurring in healthy individuals. Participants were given a set of multiple-choice questions with options such as “Yes,” “No,” or “I don’t know,” and in some cases, specific diagnostic or microbial options.

To assess the level of knowledge among participants, each correct answer was assigned a score of one (1), and incorrect or “I don’t know” responses were assigned a score of zero (0). A total knowledge score ranging from 0 to 5 was computed for each respondent by summing the scores of five key questions. Participants who correctly answered more than 70% of the knowledge questions (a score of 4 or 5 out of 5) were categorized as having good knowledge. Those scoring between 40% and 70% (scores of 2 or 3) were considered to have average knowledge, while scores below 40% (0 or 1) indicated poor knowledge.

The questionnaire was available in both Arabic and English. Prior to data analysis, responses were reviewed for completeness and accuracy. The instrument underwent both content and face validation by clinical experts to ensure clarity, relevance, and appropriateness of the questions.

Data analysis

The collected data were analyzed using SPSS version 26 (IBM Corp., Armonk, NY). Descriptive statistics (frequencies and percentages) were used to summarize categorical data. The chi-square test was employed to assess associations between demographic characteristics and awareness levels. A p-value of <0.05 was considered statistically significant.

Ethical considerations

Informed Consent

All participants provided informed consent before participation. The consent form outlined the study's purpose, procedures, potential risks, and benefits.

Confidentiality

Participants' identities were anonymized using unique identification codes, and no personally identifiable information was collected. Data were securely stored on password-protected devices accessible only to the research team.

Ethical Approval

This study was reviewed and approved by AlMaarefa University Research Ethics Committee under the IRB number IRB25-008.

## Results

A total of 106 participants completed the survey. The majority were female (n = 62, 58.5%), while males accounted for 41.5% (n = 44). Most respondents were Saudi nationals (n = 87, 82.1%), with non-Saudis comprising 17.9% (n = 19). The largest proportion of participants was within the 18-29 years age group (n = 62, 58.5%), followed by 30-39 years (n = 28, 26.4%), 40-49 years (n = 13, 12.3%), and 50 years or older (n = 3, 2.8%). A substantial number were single (n = 84, 79.2%), while 20.8% (n = 22) were married. Regarding educational attainment, most participants held a bachelor’s degree (n = 83, 78.3%), followed by postgraduate qualifications (n = 16, 15.1%) and secondary school education (n = 7, 6.6%). In terms of occupation, healthcare providers represented the largest group (n = 40, 37.7%), followed by individuals classified under other professions (n = 30, 28.3%), teachers (n = 12, 11.3%), engineers (n = 10, 9.4%), military personnel (n = 7, 6.6%), and students (n = 7, 6.6%) (Table 1).

**Table 1:** Sociodemographic characteristics of study participants (N = 106). This table presents the distribution of participants according to gender, nationality, age, marital status, educational level, and occupation. Frequencies and percentages are reported for each category.

Parameter		Frequency	Percentage
Gender	Male	44	41.5
	Female	62	58.5
Nationality	Saudi	87	82.1
	Non-Saudi	19	17.9
Age	18-29	62	58.5
	30-39	28	26.4
	40-49	13	12.3
	50+	3	2.8
Marital status	Single	84	79.2
	Married	22	20.8
Education	Secondary school	7	6.6
	Bachelor	83	78.3
	Postgraduate	16	15.1
Occupation	Student	7	6.6
	Teacher	12	11.3
	Military	7	6.6
	Healthcare provider	40	37.7
	Engineer	10	9.4
	Others	30	28.3

With respect to knowledge, the majority of participants (n = 74, 69.8%) reported having heard of NF. Sixty-five respondents (61.3%) correctly identified bacterial infection as the primary cause of the disease, and 48 participants (45.3%) recognized group A *Streptococcus* as a commonly implicated organism. When asked about the speed of disease progression, 44.3% (n = 47) correctly indicated that symptoms can develop within hours. Additionally, 54 participants (50.9%) were aware that NF can occur even in the absence of traditional risk factors. Regarding attitudes, the vast majority (n = 75, 70.8%) emphasized the importance of early treatment in improving outcomes, and an equal proportion (n = 75, 70.8%) endorsed the need for public education and awareness initiatives. Most participants (n = 86, 81.1%) expressed interest in obtaining more information about the disease. In terms of management perceptions, surgical intervention was most frequently identified as the appropriate treatment option (n = 64, 60.4%), while proper wound care was the most commonly reported preventive measure (n = 54, 50.9%). A majority of participants reported first learning about the condition from medical professionals (n = 58, 54.7%), whereas 32 individuals (30.2%) had never heard of the condition before participating in the survey (Table 2).

**Table 2:** Participants’ knowledge, attitudes, and practices regarding necrotizing fasciitis. This table summarizes responses to key questions assessing participants’ awareness, understanding, and perceptions related to necrotizing fasciitis. Items are categorized into knowledge, attitudes, and practices. Responses are shown as frequencies and percentages.

Parameter		Options	Frequency (%)
Knowledge	Have you heard of necrotizing fasciitis before?	Yes	74 (69.8)
		No	32 (30.2)
	What do you think causes necrotizing fasciitis?	Bacterial infection	65 (61.3)
		Viral infection	10 (9.4)
		Fungal infection	8 (7.5)
		I don't know	23 (21.7)
	Which bacteria are commonly associated with necrotizing fasciitis?	Group A Streptococcus	48 (45.3)
		Staphylococcus aureus	22 (20.8)
		Escherichia coli	5 (4.7)
		I don't know	31 (29.2)
	How would you rate your awareness of necrotizing fasciitis on a scale from 1 (Unaware) to 4 (Highly aware)?	1. Unaware	33 (31.1)
		2. Partially aware	4 (3.8)
		3. Somewhat aware	68 (64.2)
		4. Highly aware	1 (0.9)
	How fast do you believe necrotizing fasciitis symptoms progress?	Within hours	47 (44.3)
		Within days	23 (21.7)
		Within weeks	5 (4.7)
		I don't know	31 (29.2)
	What are the risk factors for developing necrotizing fasciitis?	Cuts or wounds	24 (22.6)
		Diabetes	14 (13.2)
		Weak immune system	28 (26.4)
		Recent surgery	11 (10.4)
		I don't know	29 (27.4)
	Can necrotizing fasciitis occur in healthy individuals without risk factors?	Yes	54 (50.9)
		No	52 (49.1)
Attitude	How important is early treatment for necrotizing fasciitis outcomes?	Very important	75 (70.8)
		Somewhat important	6 (5.7)
		Not important	5 (4.7)
		I don't know	20 (18.9)
	Do you think public education and awareness about necrotizing fasciitis are necessary?	Yes	75 (70.8)
		No	6 (5.7)
		I’m not sure	25 (23.6)
	Would you like more information about necrotizing fasciitis?	Yes	86 (81.1)
		No	20 (18.9)
Practice	What type of treatment do you think is needed for necrotizing fasciitis?	Antibiotics	12 (11.3)
		Surgical	64 (60.4)
		Painkillers	3 (2.8)
		I don't know	27 (25.5)
	How can necrotizing fasciitis be prevented?	Proper wound care	54 (50.9)
		Vaccination	6 (5.7)
		Avoiding contact	5 (4.7)
		Good hygiene	15 (14.2)
		I don't know	26 (24.4)
	Where did you first hear about necrotizing fasciitis?	Internet	12 (11.3)
		Television	1 (0.9)
		Medical professional	58 (54.7)
		Family or friends	3 (2.8)
		Never heard about it	32 (30.2)

Analysis of the association between sociodemographic characteristics and knowledge level revealed several statistically significant findings. A significantly higher proportion of females demonstrated good knowledge compared to males (79.2% vs. 20.8%, p = 0.001, χ² = 15.45). Age was also significantly associated with knowledge level (p = 0.009, χ² = 11.696), with participants aged 40-49 years showing a higher proportion of good knowledge (22.9%) relative to other age groups. Education level was significantly associated with knowledge (p = 0.009, χ² = 9.366); notably, none of the participants with secondary school education demonstrated good knowledge, whereas 22.9% of those with postgraduate qualifications did. Occupational status was also significantly correlated with knowledge (p = 0.001, χ² = 30.663). Half of the healthcare providers (50%) exhibited good knowledge, in contrast to none among students, teachers, or military personnel. No statistically significant associations were found between knowledge level and either nationality (p = 0.223) or marital status (p = 0.643) (Table 3).

**Table 3:** Association between sociodemographic factors and knowledge level about necrotizing fasciitis. Data are presented as frequency (n) and percentage (%). Chi-square test was used to assess associations. A p-value of <0.05 was considered statistically significant.

		Knowledge			
Parameter		Good	Poor	P-value	Chi square
Gender	Male	10 (20.8)	34 (58.6)	0.001	15.45
	Female	38 (79.2)	24 (41.4)		
Nationality	Saudi	37 (77.1)	50 (86.2)	0.223	1.486
	Non-Saudi	11 (22.9)	8 (13.8)		
Age	18-29	24 (50)	38 (65.5)	0.009	11.696
	30-39	13 (27.1)	15 (25.9)		
	40-49	11 (22.9)	2 (3.4)		
	50+	0	3 (5.2)		
Marital status	Single	39 (81.3)	45 (77.6)	0.643	0.214
	Married	9 (18.8)	13 (22.4)		
Education	Secondary school	0	7 (12.1)	0.009	9.366
	Bachelor	37 (77.1)	46 (79.3)		
	Postgraduate	11 (22.9)	5 (8.6)		
Occupation	Student	0	7 (12.1)	0.001	30.663
	Teacher	0	12 (20.7)		
	Military	0	7 (12.1)		
	Healthcare provider	24 (50)	16 (27.6)		
	Engineer	4 (8.3)	6 (10.3)		
	Others	20 (41.7)	10 (17.2)		

## Discussion

This study aimed to assess the level of awareness and knowledge of NF among the general population and healthcare workers in Riyadh, Saudi Arabia. Overall, the findings reflect moderate awareness but significant knowledge gaps, particularly in understanding risk factors, disease progression, and management. These findings are in line with existing literature highlighting NF as a frequently misdiagnosed and poorly recognized condition in its early stages [1,2].

A notable observation was that only 61.3% of participants correctly identified bacterial infection as the underlying cause of NF, and less than half (45.3%) recognized group A *Streptococcus* as a commonly implicated organism. This corresponds with findings by Puvanendran et al. [2], who emphasized the general under-recognition of the bacterial etiology of NF, particularly among non-clinicians. Similarly, Mohammed [1] found that even among physicians in Saudi Arabia, misconceptions about the causative agents of NF persisted, further underscoring this knowledge gap.

In our study, 44.3% of participants correctly identified the rapid progression of symptoms, which aligns with previous findings that many individuals fail to appreciate the acute onset of NF. Andersson et al. [3] demonstrated through a multi-center qualitative analysis that patients often underestimated the speed at which NF advances, frequently delaying presentation to healthcare services. This delay in seeking care contributes to poor prognosis and greater morbidity, as highlighted in the review by Anaya and Dellinger [10].

The level of awareness surrounding NF risk factors was varied, with just over half of respondents recognizing that the disease can occur in otherwise healthy individuals. This misconception echoes findings by Fais et al. [5], who noted that both patients and clinicians often associate NF exclusively with high-risk individuals, leading to diagnostic delays in atypical presentations. A recent meta-analysis confirmed that comorbidities such as diabetes and immunosuppression significantly increase mortality, while healthy individuals are still at risk when diagnosis is delayed [12,13].

Encouragingly, a majority of participants (70.8%) recognized the importance of early treatment, aligning with established evidence emphasizing the urgency of prompt surgical and antibiotic intervention to reduce mortality [4,6,9]. More recent reviews corroborate these findings, showing that operative debridement within the first 12 hours can reduce case fatality rates by up to 40% [12].

Regarding sources of information, more than half of the respondents learned about NF through medical professionals (54.7%). This contrasts with findings by Alvarez Hernández et al. [7], where the majority of participants obtained their knowledge through digital platforms, reflecting regional differences in information access. Community-level awareness campaigns have been shown to shorten time to presentation and improve survival in endemic areas [14].

Our data also revealed significant associations between knowledge levels and sociodemographic characteristics, notably gender, age, education level, and occupation. Females demonstrated significantly higher knowledge scores than males, a trend previously observed in community-based infectious disease research [8]. Additionally, healthcare providers exhibited significantly greater knowledge compared with other occupational groups, consistent with the positive correlation between clinical exposure and disease understanding reported by Alshammari et al. [9].

Although the LRINEC score is widely used, its predictive accuracy remains controversial; modified versions have demonstrated improved sensitivity for mortality prediction [15].

This study has several limitations that should be acknowledged. First, the cross-sectional design captures participants’ knowledge and perceptions at a single point in time, limiting causal inference. Second, although the sample included both healthcare professionals and members of the general public, it was confined to Riyadh, Saudi Arabia, which may restrict generalizability to other regions with differing healthcare access profiles. Third, reliance on self-reported data introduces recall and social desirability bias. Finally, the binary classification of knowledge (good vs. poor) based on an arbitrary cut-off may oversimplify the continuum of understanding. Future research should incorporate psychometric validation of instruments, employ longitudinal designs, and explore the impact of targeted educational interventions on diagnostic delay and outcomes.

## Conclusions

This study highlights a moderate level of awareness but limited in-depth knowledge of NF among participants in Riyadh, with notable gaps in recognizing its rapid progression, causative organisms, and risk factors. While healthcare providers demonstrated relatively higher knowledge levels, misconceptions were still evident across all demographic groups. The significant associations observed between knowledge levels and factors such as gender, education, age, and occupation underscore the need for targeted educational interventions. Enhancing public and professional understanding of NF is essential for improving early detection, timely management, and ultimately reducing the high morbidity and mortality associated with this aggressive condition. Future efforts should prioritize community awareness campaigns, continuing medical education, and the integration of critical infectious disease topics into public health curricula.
